# The Presence of Type Va Double Common Bile Duct in a Male Patient: A Case Report

**DOI:** 10.7759/cureus.72801

**Published:** 2024-10-31

**Authors:** Jamie McDermott, Shakira E Gonzalez, Zurisadai Medina Alonso, Julio A Oquendo Figueroa, April Nunez, Imtiaz Ahmed

**Affiliations:** 1 Medicine, Midwestern University Arizona College of Osteopathic Medicine, Glendale, USA; 2 Medicine, Universidad Autonoma de Guadalajara, Guadalajara, MEX; 3 Department of Radiology, Tempe St. Luke's Hospital, Tempe, USA

**Keywords:** acute calculus cholecystitis, cholelithiasis, common bile duct, common bile duplication, double common bile duct

## Abstract

Double common bile duct (DCBD) is a rare congenital anomaly of the biliary system, characterized by the presence of two common bile ducts. The condition can be classified into five distinct types. Type Va DCBD is one of the rare congenital variations where both bile ducts open separately into the duodenum: one opens normally at the major duodenal papilla, and the second opens independently into the duodenum at a different site, usually proximal to the major duodenal papilla. DCBD is often associated with biliary pathologies such as cholelithiasis, choledocholithiasis, choledochal cysts, abnormal pancreaticobiliary junction (APBJ), and upper gastrointestinal malignancies. We present the case of an 83-year-old male from the United States who presented to the emergency department with right upper quadrant (RUQ) pain. Magnetic resonance cholangiopancreatography (MRCP) and endoscopic retrograde cholangiopancreatography (ERCP) revealed a type Va DCBD, complicated by cholelithiasis and acute calculous cholecystitis. The patient underwent a laparoscopic cholecystectomy, along with common bile duct stent placement for biliary drainage. This case highlights the importance of identifying atypical radiologic features indicative of rare congenital biliary anomalies such as type Va DCBD. Given the scarcity of reported cases in the literature, accurate preoperative imaging and early diagnosis of this anomaly are crucial for preventing long-term complications and guiding appropriate surgical management.

## Introduction

The duplication of the bile duct or double common bile duct (DCBD) is a rare congenital anomaly characterized by the presence of either a septum within the common bile duct or two distinct bile ducts [1]. Embryogenesis of the gallbladder, biliary tree, and liver begins during the third to fourth week of gestation [2]. It starts with the formation of the hepatic diverticulum, which is subsequently divided into the ventral and dorsal buds. The ventral bud gives rise to the primitive gallbladder, while the dorsal bud develops into the right and left lobes of the liver. As the biliary tree continues to mature, the hepatic duct undergoes significant transformation. The formation of the biliary tree lumen results from the proliferation and vacuolization of epithelial cells, leading to the development of two distinct channels within the bile duct. 

DCBD is classified into five subtypes. According to the literature, only six cases of type Va DCBD were reported between 2007 and 2017 [1]. All reported cases involved female patients of various ages ranging from late 20s to over 60 years old. These cases were identified in various places such as Asia, Ireland, and Mexico. There have been no reported cases of type Va DCBD in the United States [1]. 

Given the limited number of cases in the literature, understanding normal biliary anatomy is critical for accurate diagnosis. The normal anatomy of the bile duct facilitates drainage into the duodenum. In cases of a DCBD, an additional bile duct, referred to as the accessory common bile duct (ACBD), may open into various segments of the upper gastrointestinal tract, including the stomach, duodenum, pancreatic duct, and septum. The ACBD drains bile from different regions of the liver, which raises significant clinical concerns. A DCBD can be dangerous for several reasons, primarily due to the abnormal anatomy and the associated complications that may arise. For example, the presence of two bile ducts can increase the likelihood of bile duct stones (choledocholithiasis), which can obstruct bile flow. DCBD has also been linked with an increased risk of malignancies in the biliary system or upper gastrointestinal tract due to chronic inflammation and bile stasis [1,3,4]. 

During surgery (e.g., cholecystectomy), surgeons may inadvertently damage the duplicated bile ducts if they are unaware of the anomaly, leading to bile leakage or stricture formation, which can have long-term consequences, including the need for additional surgeries. Treatments for this condition include endoscopic retrograde cholangiopancreatography (ERCP), surgery, or laparoscopic cholecystectomy [1]. The presence of a DCBD represents a critical anatomical variant that may predispose patients to various complications, including choledocholithiasis, cholangitis, pancreatitis, and malignancies of the upper gastrointestinal tract.

## Case presentation

We present an 83-year-old male from the United States with a medical history significant for hypertension and type 2 diabetes mellitus, who presented to the emergency department with complaints of substernal chest pain, shortness of breath, and right upper quadrant (RUQ) pain onset two days ago. Upon admission, the physical examination revealed severe tenderness in the RUQ, along with a positive Murphy’s sign.

**Figure 1:**
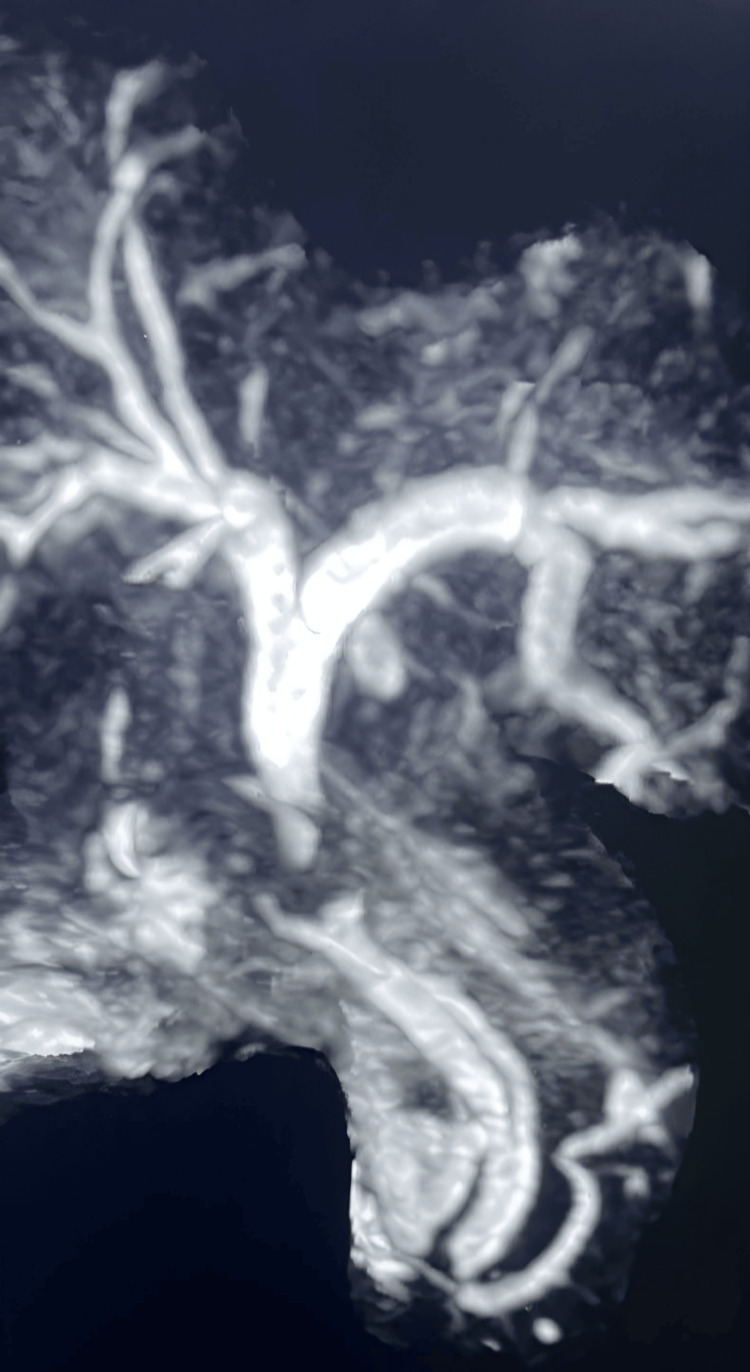
MRCP image of the patient Magnetic resonance cholangiopancreatography (MRCP) reveals the presence of double common bile ducts. A type Va DCBD is one of the rare congenital variations where both bile ducts open separately into the duodenum: one opens normally at the major duodenal papilla and the second opens independently into the duodenum at a different site, usually proximal to the major duodenal papilla.

Several imaging modalities were employed for further evaluation. An abdominal ultrasound (US) of the RUQ identified a 19 mm fixed gallstone within the gallbladder, visible pericholecystic fluid, and no evidence of gallbladder wall thickening. A computed tomography (CT) angiography of the chest confirmed the presence of cholelithiasis. The CBD measured 4 mm, with no stones or dilation noted. MRCP further revealed a contracted gallbladder containing multiple gallstones, indicative of acute calculous cholecystitis, and a large stone lodged in the neck of the gallbladder, causing a mass effect on the CBD (Figure 1).

**Figure 2:**
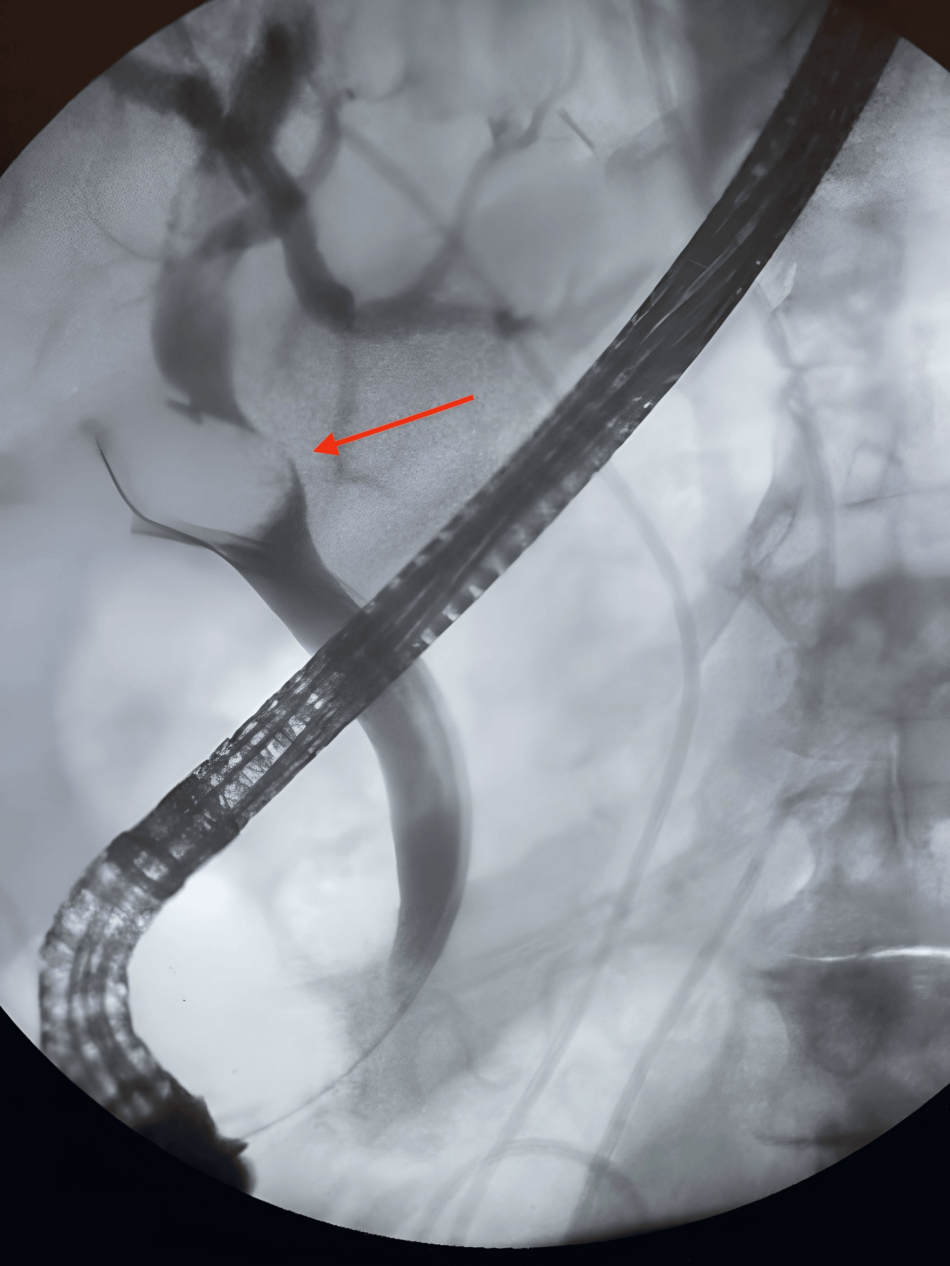
ERCP image of the patient Endoscopic retrograde cholangiopancreatography (ERCP) demonstrates a large filling defect (red arrow) at the junction of the cystic duct and common bile duct, indicative of a retained large stone.

No evidence of choledocholithiasis was observed, but a duplication of the CBD was noted, suggestive of a type Va DCBD. Subsequently, a biliary and pancreatic ERCP was performed, which demonstrated a significant filling defect at the junction of the cystic duct and CBD, consistent with the presence of a large stone. After the patient underwent cholecystectomy and placement of a CBD stent, a follow-up ERCP revealed the stent in position, but the CBD was poorly visualized (Figure 2). 

Following initial treatment, an immediate postoperative follow-up plan was established, including an outpatient ERCP within six to eight weeks to evaluate the common bile duct (CBD) for residual stones, confirm ductal patency, and assess the CBD stent placement. Stent removal will be considered if no stones or obstructions are identified. Additionally, an in-person follow-up appointment is scheduled within two to four weeks post-discharge to evaluate for any new symptoms and to monitor for potential complications, such as infection or bile duct injury. There has been no recurrence of symptoms observed at the time of writing.

## Discussion

Duplication of the common bile duct (DCBD) is an exceedingly rare anomaly arising from the incomplete regression of the embryological double biliary system, first documented in 1543 [5]. Classified into five subtypes, type Va is the rarest [6,7]. The current classification of double common bile ducts is based on morphological criteria allowing for the recognition of type V variants, whereas the initial classification relied on anatomical appearance [5]. This case of type Va DCBD, complicated by cholelithiasis and acute calculous cholecystitis, demonstrates the importance of detailed anatomical understanding in surgical planning to avoid bile duct injury. Preoperative imaging, such as MRCP and ERCP, is crucial to identifying DCBD and preventing misdiagnosis or delayed diagnosis, which can lead to serious complications. However, DCBD is challenging to detect even with imaging, often resulting in overlooked or misdiagnosed cases. Adequate preoperative imaging helps ensure safer operative management, given the high risk of ductal injury and postoperative complications. In this case, cholecystectomy and CBD stenting were chosen to manage symptoms, reflecting the need to tailor interventions based on the DCBD subtype and associated risks. Multiple reports advocate for the resection of accessory or extrahepatic bile ducts due to the potential risks of recurrent cholangitis and carcinogenesis associated with ectopic drainage sites; however, the long-term outcomes of this approach remain uncertain [8].

Patients with DCBD are predisposed to complications such as bile duct obstruction, inflammation, and elevated long-term cancer risks due to altered anatomical flow, particularly in the presence of an anomalous pancreaticobiliary junction. For type Va DCBD cases without malignancy, accessory bile duct resection is recommended to minimize long-term risks. Recognizing the embryological basis of these malformations underscores the potential for complex sequelae throughout life, and the literature suggests a significant correlation with heightened risks for other biliary or pancreatic conditions, necessitating close monitoring [9,10].

This case may suggest a potential ethnic or geographic factor influencing the presentation of type Va DCBD, as previous reports predominantly involve female patients from Asia, Ireland, and Mexico. Future research should explore whether genetic, environmental, or healthcare access disparities contribute to this anomaly’s frequency and presentation in these populations, as well as investigate any regional differences in diagnostic approaches and outcomes.

## Conclusions

This case highlights the necessity of recognizing atypical radiologic findings indicative of rare congenital anomalies, such as a type Va double common bile duct (DCBD). Given the limited literature on type Va DCBD, adequate preoperative imaging and early diagnosis are crucial in preventing long-term complications and in guiding appropriate surgical intervention. This rare case of type Va DCBD underscores the importance of detailed biliary anatomy knowledge, appropriate imaging, and awareness of potential complications. Additionally, gender, age, and geographical factors, such as healthcare access and diagnostic resources, may influence the timeliness and accuracy of diagnosis, ultimately affecting patient outcomes and management. Notably, we believe this is the first reported case of type Va DCBD in the United States. Previous cases have predominantly involved female patients from regions outside the United States and varying age groups, making this case particularly distinct. This case not only contributes to the understanding of type Va DCBD but also highlights the need for heightened clinical awareness and tailored approaches in diagnosing and managing such rare anomalies across diverse patient demographics.
